# Delayed-Onset Post-extubation Laryngeal Edema Complicated by Epiglottitis: A Case Report

**DOI:** 10.7759/cureus.82144

**Published:** 2025-04-12

**Authors:** Jeffrey Valencia Uribe, Cecilia Nosti, Suset Almuinas de Armas, Christopher Day, Yamil Selman, Benjamin T Houseman, Dahlia Blake

**Affiliations:** 1 Internal Medicine, Memorial Healthcare System, Pembroke Pines, USA; 2 Anesthesiology, Memorial Healthcare System, Pembroke Pines, USA; 3 Head and Neck Surgical Oncology, Memorial Healthcare System, Pembroke Pines, USA; 4 Anesthesiology, Envision Physician Services, Pembroke Pines, USA; 5 Critical Care Medicine, Memorial Healthcare System, Pembroke Pines, USA

**Keywords:** airway management, epiglottitis, laryngoscopy, post-extubation laryngeal edema, unilateral arytenoid swelling

## Abstract

Post-extubation laryngeal edema (PELE) is a well-documented complication of endotracheal intubation, typically presenting within hours of extubation. PELE, following elective procedures, is usually mild and resolves with conservative management. We report the case of a 73-year-old male patient who developed progressive odynophagia and dysphagia 24 hours after an uneventful inguinal hernia repair under general anesthesia. A contrast-enhanced CT scan of the neck revealed laryngeal edema extending to the epiglottis, raising concern for epiglottitis. Flexible fiberoptic laryngoscopy confirmed moderate edema involving the arytenoids and epiglottis, with preservation of airway patency. The patient was treated empirically with corticosteroids and broad-spectrum antibiotics due to concern for a possible infectious process, and subsequently made a full recovery without recurrence of symptoms.

This case illustrates an unusual presentation of delayed-onset PELE coexisting with epiglottitis in the absence of a confirmed infectious etiology. It underscores the importance of early recognition and careful airway monitoring in patients with evolving post-extubation symptoms. While corticosteroids are effective in reducing PELE incidence, their role in treating epiglottitis remains debated due to inconsistent evidence regarding benefits and potential risks. Further investigation is necessary to clarify the association between endotracheal intubation and the development of epiglottitis, and to inform evidence-based approaches for managing cases where PELE and supraglottic infection overlap.

## Introduction

Post-extubation laryngeal edema (PELE) is a well-recognized complication of endotracheal intubation, with reported incidence rates ranging from 5% to over 54% depending on patient population and diagnostic criteria [1,2]. PELE typically presents within hours of extubation and is characterized by mild symptoms such as hoarseness, sore throat, dysphagia, and odynophagia; however, in more severe cases, it may progress to stridor and respiratory distress. In contrast, both infectious and non-infectious epiglottitis tend to present more acutely and severely, often with stridor, drooling, sore throat, and respiratory distress. Infectious epiglottitis is typically accompanied by high fever and systemic signs, while non-infectious forms lack these systemic features. The underlying pathophysiology of PELE involves inflammation and edema of the arytenoid cartilages, vocal cords, cricoarytenoid joints, posterior glottis, and subglottic regions [1,2]. Epiglottitis, characterized by inflammation of the supraglottic region, is an atypical and rare post-extubation complication that is not well documented in the literature.

This case highlights the diagnostic and therapeutic challenges of managing a patient with delayed-onset PELE complicated by concurrent epiglottitis, a rare and clinically complex presentation that blurs the line between the two conditions and necessitates a high degree of clinical vigilance.

## Case presentation

A 73-year-old male patient with a medical history of asthma, gastroesophageal reflux disease (GERD), prior tonsillectomy, dyslipidemia, and occasional tobacco smoking presented to the emergency department with complaints of difficulty swallowing one day after undergoing an uncomplicated elective inguinal hernia repair under general anesthesia with endotracheal intubation.

Preoperatively, the patient’s airway assessment revealed a Mallampati classification of three, a thyromental distance greater than three fingerbreadths, and a full neck range of motion. In the pre-operative holding area, the patient received dexamethasone (4 mg IV), famotidine (20 mg IV), acetaminophen (1000 mg by mouth), and midazolam (1 mg IV). General anesthesia was induced, and endotracheal intubation was performed uneventfully using a Macintosh 3 laryngoscope with a cuffed 7.5 mm endotracheal tube. Subsequently, an orogastric tube was inserted, and cefazolin (2 g IV) was administered for surgical site infection prophylaxis.

On postoperative day 1, the patient arrived at the emergency department in mild distress due to odynophagia and difficulty swallowing solids. Vital signs were unremarkable except for a mild fever (38.5°C). Physical examination of the mouth and posterior pharynx was unremarkable, but submandibular tenderness was noted on palpation. Cardiac and pulmonary examinations were normal. Laboratory results revealed neutrophilic leukocytosis, white blood cell 14.3 x 10^3^/µL (reference range: 4-11.0 x 10^3^/µL), an elevated C-reactive protein level of 3.11 mg/dL (reference range: <0.5 mg/dL), and procalcitonin of 2.52 ng/mL (reference range: <0.1 ng/mL), supporting an underlying inflammatory or infectious process.

**Figure 1:**
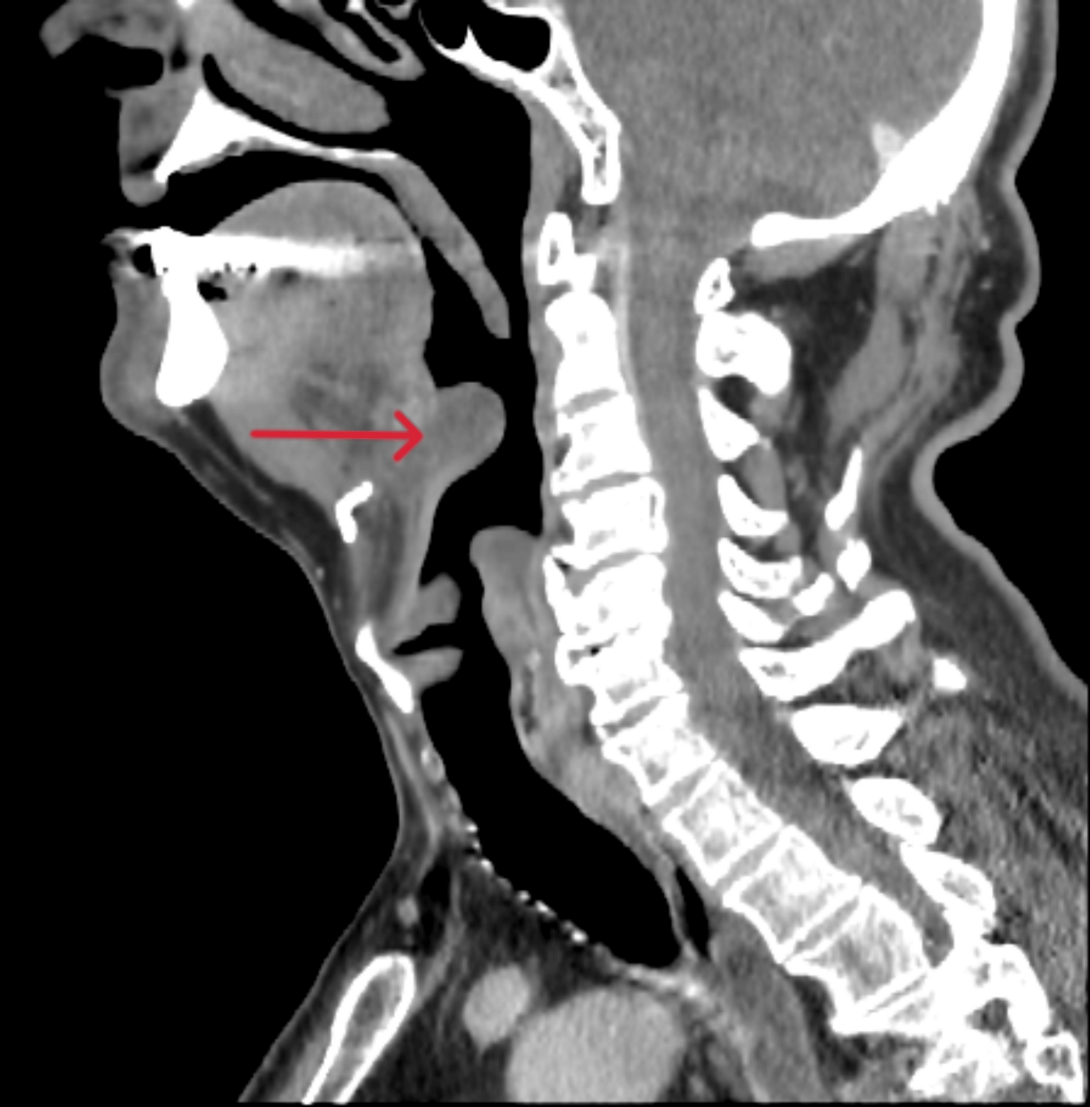
A CT scan of the neck soft tissue with intravenous contrast revealed mild to moderate laryngeal edema extending superiorly and involving the epiglottis (red arrow), concerning for epiglottitis versus iatrogenic from recent instrumentation. There was no evidence of airway stenosis.

Infectious workups, including COVID-19, influenza, respiratory syncytial virus, and blood cultures, were negative. A contrast-enhanced CT scan of the neck (Figure 1) revealed mild to moderate laryngeal edema extending superiorly and involving the epiglottis, without evidence of airway stenosis. The vocal cords appeared symmetric. The differential diagnosis included edema from recent airway instrumentation or epiglottitis. Given concern for an infectious etiology, the patient was started empirically on ceftriaxone, vancomycin, and dexamethasone 8 mg IV every eight hours for 24 hours to reduce airway inflammation.

**Figure 2:**
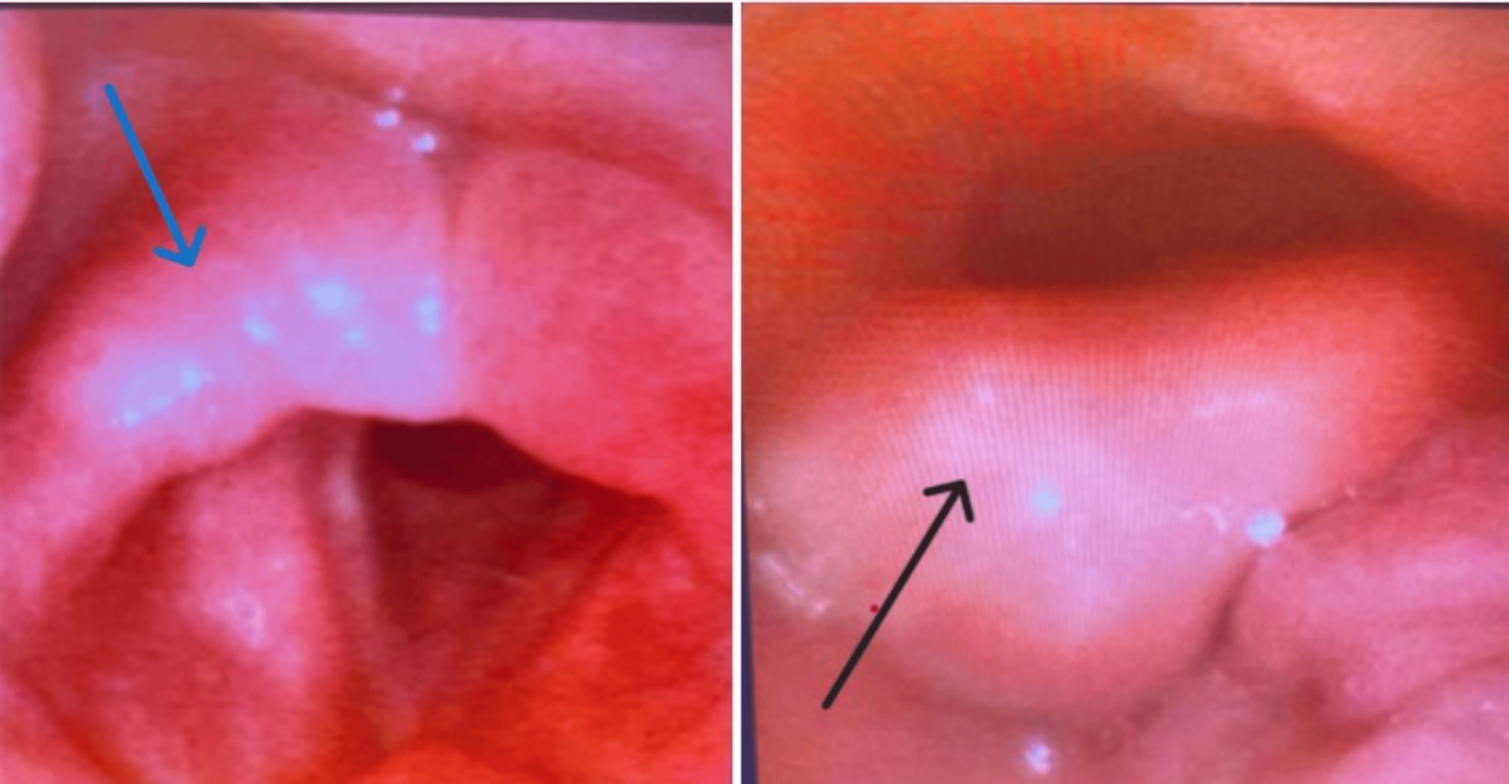
Fiberoptic laryngoscopy demonstrated increased secretions with minimal pooling and moderate laryngeal edema, predominantly affecting the arytenoids (more pronounced on the right (blue arrow) than the left) and epiglottis (black arrow). The airway was patent, with no evidence of distinct masses, erythroplakia, or leukoplakia.

By postoperative day 3, the patient’s dysphagia had worsened, and he developed new-onset hoarseness. He was subsequently transferred to the intensive care unit (ICU) for closer monitoring of his airway. The patient maintained a reassuring oxygenation status (96% on room air) with no symptoms of respiratory distress. A swallow evaluation demonstrated pharyngeal dysphagia, prompting continued aspiration precautions. Flexible fiberoptic laryngoscopy (Figure 2) revealed increased secretions without pooling in the hypopharynx and moderate edema of the arytenoids (right greater than left) and epiglottis. Vocal fold motion was normal, with full abduction and adduction, and no masses or lesions were seen. The patient continued to improve clinically during his ICU stay, tolerating thickened liquids and gradually regaining his voice quality.

**Figure 3:**
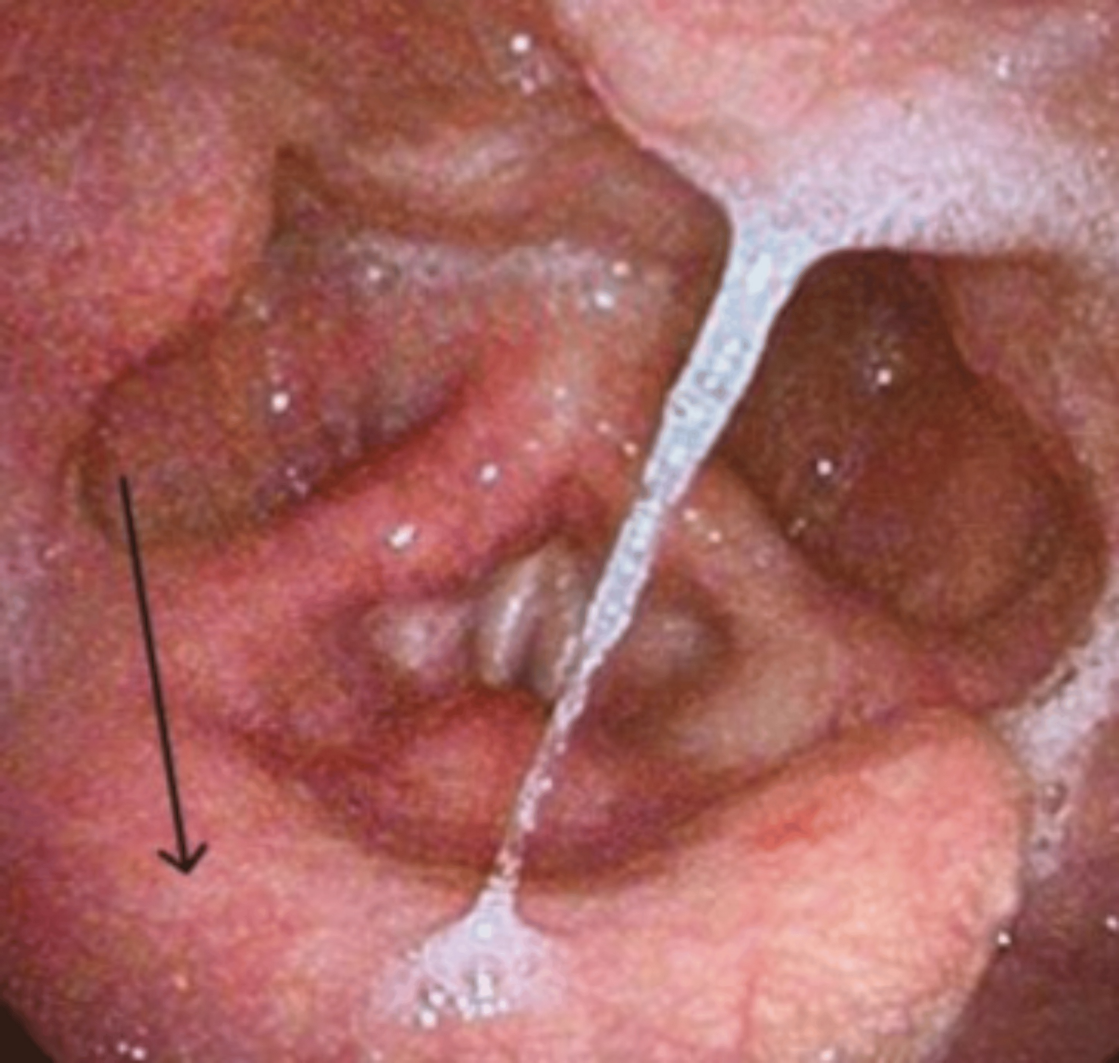
Repeat laryngoscopy revealed normal vocal cord motion, mild erythema of the epiglottis (black arrow), and cobblestoning of the hypopharyngeal mucosa, consistent with resolving epiglottitis and laryngopharyngeal reflux.

He was discharged on a Medrol Dosepak and amoxicillin-clavulanic acid, both of which he completed. At the two-week follow-up, the patient reported complete resolution of odynophagia and fever. Residual symptoms included mild, intermittent dysphagia with liquids, which improved with throat clearing. A repeat laryngoscopy (Figure 3) demonstrated normal vocal cord motion, mild erythema of the epiglottis, and cobblestoning of the hypopharyngeal mucosa, consistent with resolving epiglottitis and laryngopharyngeal reflux. The patient was prescribed omeprazole for ongoing management and was scheduled for a second follow-up evaluation with the otolaryngology team to monitor symptom resolution and ensure continued clinical improvement.

## Discussion

Clinical presentation and diagnostic considerations

PELE is typically a mild, self-limiting condition that develops within hours following extubation. In a prospective study by François et al., 12% of patients required reintubation due to laryngeal edema, with the majority developing symptoms within 30 minutes and nearly half within five minutes post-extubation [3]. Although our patient had several risk factors for airway complications, including a Mallampati score of three and a history of asthma, GERD, and tobacco use, the onset of symptoms approximately 24 hours after extubation was unusual, particularly in the context of preoperative dexamethasone administration, which is known to reduce the risk of airway edema [3].

Progressive dysphagia and new-onset hoarseness, despite empiric treatment with antibiotics and corticosteroids, raised concern for a more complex process than typical PELE. Contrast-enhanced CT revealed mild to moderate laryngeal edema extending superiorly to involve the epiglottis, raising suspicion for concurrent epiglottitis. Distinguishing PELE from epiglottitis can be challenging due to overlapping symptoms such as dysphagia, hoarseness, and odynophagia. However, systemic features like fever and a toxic appearance are more characteristic of epiglottitis and are typically absent in PELE [4,5]. 

In this case, flexible laryngoscopy confirmed moderate edema involving the arytenoids and epiglottis without evidence of airway obstruction. Laryngeal edema may impair vocal cord mobility and increase aspiration risk; the isolated involvement of the right arytenoid and epiglottis was more consistent with localized inflammation than diffuse mechanical trauma [1]. Peritonsillar abscess (PTA), a known comorbidity in acute epiglottitis, may present with similar findings. In a retrospective study of 139 cases, PTA was observed in 15%, with inferior-type PTA often manifesting as unilateral arytenoid swelling [6]. Epiglottic involvement was likewise reported in 15% of cases [7]. In this case, however, imaging and laryngoscopic evaluation ruled out PTA, further supporting a diagnosis of epiglottitis.

Pathophysiology

Although post-intubation epiglottitis is rare, it has been reported in association with laryngeal mask airway use, particularly in cases of cuff overinflation, malposition, or prolonged duration [8,9]. Recent literature also suggests that epiglottic downfolding during intubation has been proposed as an underrecognized source of supraglottic trauma, contributing to inflammation that extends beyond the typical posterior glottic and subglottic regions affected in PELE [10].

Although epiglottitis can occur independently, its onset within 24 hours of intubation in this patient suggests a perioperative trigger. Possible contributing factors include intubation-related microtrauma, a history of tobacco use, and underlying laryngopharyngeal reflux, potentially exacerbated by mucosal irritation from preoperative glucose-rich fluid intake. In patients with compromised mucosal defenses, prolonged epithelial injury may impair healing and permit bacterial colonization, increasing the likelihood of progression from otherwise self-limiting PELE to infectious epiglottitis. The presence of fever, neutrophilic leukocytosis, and elevated procalcitonin further supported an infectious etiology. These findings suggest that a combination of airway instrumentation and patient-specific factors precipitated an inflammatory cascade leading to secondary infection rather than spontaneous epiglottitis.

Management

Corticosteroids are well-established in PELE prevention, reducing post-extubation stridor and reintubation rates [3,11-16]. However, their role in epiglottitis remains controversial; while some studies suggest benefits such as shorter ICU stays and reduced airway intervention rates, others caution that corticosteroids may mask clinical progression [17,18]. In this case, dexamethasone was administered to mitigate airway edema, with close monitoring for deterioration.

Empiric antibiotic therapy, typically penicillins or cephalosporins, was initiated to target common pathogens (*Haemophilus influenzae*, *Streptococcus pneumoniae*, Group A *Streptococcus*, *Staphylococcus aureus*), while adjunctive corticosteroids and nebulized treatments (e.g., racemic epinephrine, budesonide) are not routinely recommended [5,18-21]. Supportive strategies such as Heliox, a low-density gas mixture, can reduce turbulent airflow and airway resistance, potentially delaying the need for intubation in moderate to severe upper airway obstruction [22-24].

A conservative approach to airway management was deemed appropriate, as the patient maintained stable oxygenation throughout hospitalization and without signs of respiratory distress. Flexible fiberoptic examination confirmed a patent airway, supporting the decision to avoid intubation. As his condition improved, speech therapy helped restore his swallowing function, allowing for a gradual transition to thickened liquids. A follow-up laryngoscopy demonstrated reduced edema and normal vocal fold motion. Additionally, antireflux therapy was initiated to address a potential contribution from laryngopharyngeal reflux.

Prognosis 

The acute phase of epiglottitis typically resolves within a few days when treated appropriately, with symptoms gradually improving in parallel with decreasing inflammation on repeat laryngoscopy. When recognized early and managed effectively, the prognosis for both epiglottitis and PELE is generally favorable. Meta-analyses show that the need for airway intervention in adult epiglottitis has decreased from 20% in the 1980s to 10% by 2020, reflecting improved vaccination, early diagnosis, and noninvasive management [21,25]. Key predictors for airway intervention include epiglottic abscess, stridor, tachypnea, dyspnea, and diabetes [21,26]. Our patient lacked these high-risk features, contributing to a favorable outcome.

Although adverse events requiring respiratory support can lead to prolonged symptoms and complications in up to 15% of cases, timely management remains critical. In one study, all 61 patients survived despite 21% needing airway intervention and 62% requiring ICU admission [17, 18]. Importantly, reintubation significantly worsens outcomes, increasing mortality from 12% to 43%, and is associated with longer hospital stays and a greater need for rehabilitation. Extubation failure remains a key predictor of poor prognosis and prolonged ICU stay [27].

## Conclusions

PELE is typically a self-limiting condition; however, delayed-onset presentations should prompt thorough evaluation to rule out more serious conditions, such as infectious epiglottitis, which may require timely and aggressive management. Clinicians should maintain a high index of suspicion for epiglottitis in patients presenting with delayed PELE, particularly those with predisposing factors such as GERD, tobacco use, and worsening oropharyngeal symptoms in the presence of systemic signs.

In this case, flexible laryngoscopy was instrumental in confirming airway patency and guided the decision to pursue conservative management without intubation. When an infectious etiology is suspected, empiric antibiotic therapy should be initiated promptly. Although the role of corticosteroids in epiglottitis remains debated, clinical improvement was observed following their administration in our patient. A multidisciplinary approach, including supportive care such as speech therapy, dietary modification, and antireflux management, facilitated complete recovery and minimized complications. In addition, outpatient follow-up with laryngoscopy within one to two weeks is recommended, particularly in the setting of persistent symptoms, to assess the resolution of laryngeal edema and epiglottitis. Given the limited literature on intubation-related epiglottitis, further research is needed to better understand whether PELE associated with airway instrumentation increases the risk of epiglottitis and to establish evidence-based treatment strategies for these complex presentations.
